# Twists and turns in *Kras*-driven tumor initiation

**DOI:** 10.18632/aging.203726

**Published:** 2021-11-29

**Authors:** Yoshiaki Maru, Yoshitaka Hippo

**Affiliations:** 1 Department of Molecular Carcinogenesis, Chiba Cancer Center Research Institute, Chiba 260-8717, Japan

**Keywords:** organoid, carcinogenesis, KRAS, mouse model, pancreatic ductal adenocarcinoma

Accumulation of genetic mutations progressively drives carcinogenesis, which is affected by the cell lineage and organ-specific microenvironment. Reflecting the diversity in such cooperation, frequently mutated genes in cancer vary significantly according to the organ wherein carcinogenesis is initiated. Notably, a subset of cancers is almost exclusively associated with a particular genetic alteration, such as *KRAS* activating mutations in pancreatic ductal adenocarcinoma (PDA). Genetically engineered mice (GEM) have been considered the gold standard as a disease model of cancer. Pancreas-specific reconstitution of mutant *Kras* in mice invariably leads to the development of pre-neoplastic lesions, pancreatic intraepithelial neoplasia (PanIN), which frequently progresses to PDA in a long latency, recapitulating the multi-step pancreatic carcinogenesis in humans.

Alternatively, we have previously established a cell-based streamlined assay to probe pro-tumorigenic genetic interactions [1]. We adopted the Matrigel-based organoid culture technique because it enables long-term and physiological propagation of tissue stem cells. By integrating lentiviral gene transduction of murine organoids and their subsequent allograft implantation in immunodeficient mice, we assessed the tumorigenicity of each oragnoid [2–4]. In most cases, the outcomes were essentially similar to those observed in GEM models, even in the absence of an organ-specific microenvironment or cellular immunity, highlighting the validity and robustness of this *ex vivo* model [5,6]. Although at least two genetic alterations are usually required for developing neoplasms, mutant *Kras* alone in the pancreatic organoid exceptionally gave rise to PanIN-like lesions in 50% of tested cases [3], confirming the critical roles of mutant *Kras* in pancreatic tumorigenesis using this model. Together with the notion that *Kras* is a *bona fide* oncogene, we had hypothesized that *Kras*, regardless of wild-type (WT) or mutant, would simply confer pancreatic organoids with advantages in both *in vitro* and *in vivo* setting. However, thorough examination of organoids at each step of this model revealed that tumor initiation in the pancreas is more complicated than initially thought.

**Figure 1:**
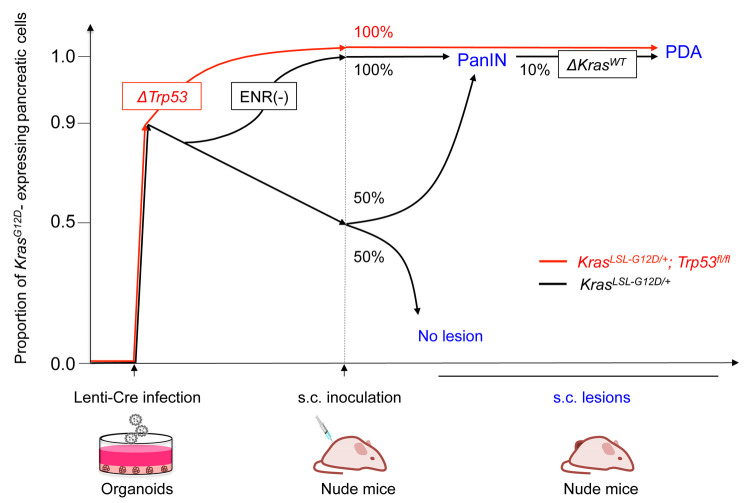
**Dynamics of the *Kras^G12D^*-expressing pancreatic cells in the organoid-based carcinogenesis model.** Pancreatic organoids from *Kras^LSL-G12D/+^* mice (black) and *Kras^LSL-G12D/+^; Trp53^fl/fl^* mice (red) were lentivirally transduced with *Cre*. Positive selection for *Kras^G12D^*-expressing cells was observed in a context-dependent manner. ERN(-), organoid culture with culture medium lacking EGF, Rsondin-1, and Noggin; PDA, pancreatic ductal adenocarcinoma; PanIN, pancreatic intraepithelial neoplasia.

In *Kras^LSL-G12D/+^* mice, transcription of *Kras^G12D^* is induced by *Cre*-mediated excision of the LoxP-Stop-LoxP (LSL) cassette. Successful *in vitro* recombination was verified by the emergence of the G12D amplicon in genomic PCR, while the retention of the LSL amplicon indicated the presence of residual cells without recombination. Given that our infection efficiency in organoids was approximately 90% [1], organoids before inoculation normally retained the LSL, although its precise quantification by regular PCR was technically challenging. In contrast, LSL was absent in tumor-derived organoids, strongly suggesting the definite requirement of *Kras^G12D^* for tumor development. To evaluate the proportion of *Kras^G12D^*-expressing cells before inoculation, we conducted puromycin selection as these cells are LSL-negative and puro-sensitive. Surprisingly, puro-sensitive cells decreased from 90% to 50% at 4 weeks after *Cre* induction (Figure 1), raising the possibility that *Kras^G12D^* could be inferior to *Kras^WT^* in the propagation of pancreatic organoids under standard culture conditions [3]. As *Kras*-activated cells are known to become less dependent on the EGF signaling pathway, we depleted EGF along with other stem cell niche factors from the medium for two weeks. As expected, *Kras^WT^* cells did not survive this harsh culture condition, leading to 100% enrichment of *Kras^G12D^*-expressing cells. In addition, the protein level of Kras^G12D^ was significantly increased after puromycin selection than anticipated by enrichment of *Kras^G12D^*-expressing cells, suggesting positive *in vitro* selection for cells with higher magnitude of Kras activation. PanIN-like lesions were induced with complete penetrance with a pure cell population with a hyperactive Kras pathway [3]. Based on these findings, we inferred that the initially observed lower tumorigenesis rate in this model than that in the GEM model might be attributable not only to the lack of an organ-specific microenvironment, but also to the underrepresentation of Kras hyper-activated cells before inoculation, which is facilitated by negative selection under standard culture conditions.

Intriguingly, the situation was observed to be different under a *p53*-null setting. After *Cre* introduction in pancreatic organoids from *Kras^LSL-G12D/+^; Trp53^fl/fl^* mice, the recombination rate quickly reached 100% for both genes, and PDA development was invariably observed, underscoring the synergy between mutant *Kras* and *p53* loss. We noted occasional PDA development from pancreatic organoids caused by *Kras^G12D^* alone, suggesting the acquisition of pro-tumorigenic alterations in the subcutis. We identified the deletion of the *Kras^WT^* allele in the PDA-derived organoid, which is also recurrently detected in human cancers carrying *KRAS* mutations. This finding was in line with the notion that *Kras^WT^* competes with *Kras^G12D^*, thereby acting as a relative tumor suppressor. Deletion of the *Kras^WT^* allele was frequently observed in *Kras*-driven tumorigenesis in the oviduct [7] and endometrial organoids [8]. These findings suggest that cells with hyperactive Kras pathway are selected in the subcutaneous tissue, in which synergistic effects of *Kras^G12D^* with the loss of *Trp53* and *Kras^WT^* provides a prominent growth advantage. It depended on organs whether *Kras^G12D^*-expressing cells were selected or declined, pointing toward the notion that carcinogenesis-related research must be conducted on the basis of organs and genes.

Although PDA development is highly dependent on activating mutations in *KRAS*, we showed that this is not a straightforward process. We uncovered anti-tumorigenic effects of both *Kras^WT^* and *Kras^G12D^*, while demonstrating that *Kras^G12D^*-expressing cells are advantageous under only some stringent conditions associated with tumor initiation, or in the context of the loss of p53 or *Kras^WT^*, which mirrors tumor progression. All these findings were obtained using the *ex vivo* carcinogenesis model, thereby warranting the use of this approach and comparison with corresponding GEM, to dissect the molecular events during tumor initiation in any organ.
